# Research progress on anti-ovarian cancer mechanism of miRNA regulating tumor microenvironment

**DOI:** 10.3389/fimmu.2022.1050917

**Published:** 2022-11-10

**Authors:** MingHua Cui, YueHui Liu, Li Cheng, Tao Li, YongZhi Deng, Da Liu

**Affiliations:** ^1^ Gynecology Department, Affiliated Hospital of Changchun University of Chinese Medicine, Changchun, Jilin, China; ^2^ Laboratory Department, Affiliated Hospital of Changchun University of Chinese Medicine, Changchun, Jilin, China; ^3^ Department of Acupuncture and Massage, The Third Affiliated Hospital of Changchun University of Chinese Medicine, Changchun, Jilin, China; ^4^ School of Pharmacy, Changchun University of Chinese Medicine, Changchun, Jilin, China

**Keywords:** miRNA, tumor microenvironment, ovarian cancer, exosomes, immunology

## Abstract

Ovarian cancer is the most deadly malignancy among women, but its complex pathogenesis is unknown. Most patients with ovarian cancer have a poor prognosis due to high recurrence rates and chemotherapy resistance as well as the lack of effective early diagnostic methods. The tumor microenvironment mainly includes extracellular matrix, CAFs, tumor angiogenesis and immune-associated cells. The interaction between tumor cells and TME plays a key role in tumorigenesis, progression, metastasis and treatment, affecting tumor progression. Therefore, it is significant to find new tumor biomarkers and therapeutic targets. MicroRNAs are non-coding RNAs that post-transcriptionally regulate the expression of target genes and affect a variety of biological processes. Studies have shown that miRNAs regulate tumor development by affecting TME. In this review, we summarize the mechanisms by which miRNAs affect ovarian cancer by regulating TME and highlight the key role of miRNAs in TME, which provides new targets and theoretical basis for ovarian cancer treatment.

## Introduction

**Figure 1:**
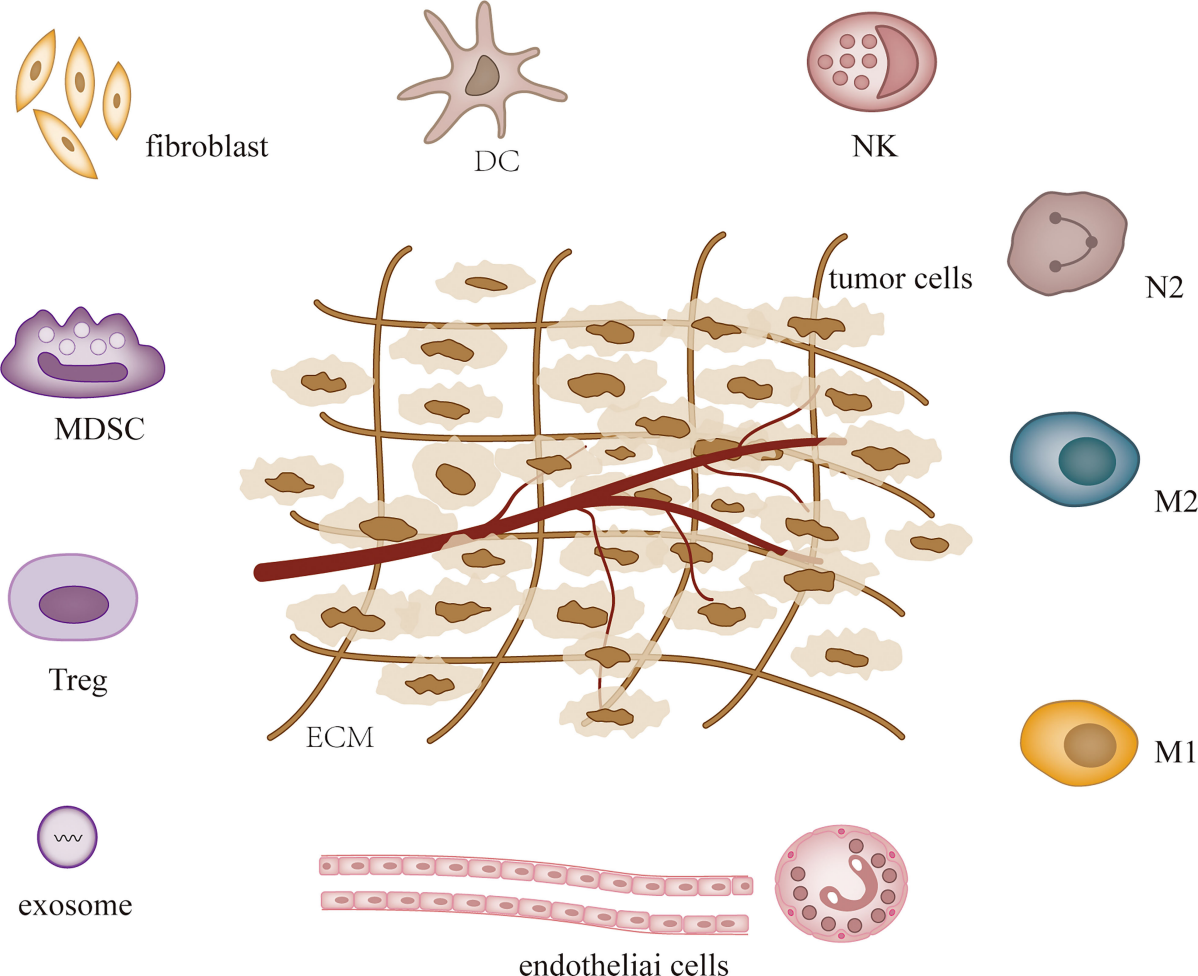
TME composition.

MicroRNAs (MiRNAs) are small non-coding RNAs that were first detected in Caenorhabditis elegans in early 1990, and since then studies have confirmed their presence in almost all species (1, 2). MiRNAs influence tumor and other disease processes by regulating post-transcriptional gene expression and participating in a variety of cellular activities (3, 4). MiRNAs are dysregulated in most tumors and the expression of specific miRNAs can characterize different tumors and stages (5, 6). Hence, miRNAs are used in the diagnosis, treatment and prognosis of cancer (7). The levels of cellular miRNAs change during tumor development, and recent studies have demonstrated that miRNAs can regulate tumor microenvironment (TME) to affect tumor angiogenesis (8, 9), immune invasion (10, 11) and tumor interstitial interactions (12, 13). TME is heterogeneous and contains a variety of cell types, including fibroblasts, endothelial cells, pericytes, immune cells, stromal stem and progenitor cells derived from local and bone marrow, and extracellular matrix (14, 15) (**Figure 1**). Some of them are altered during tumor development. Both tumor cells and their surrounding tissues influence cancer development, and TME is the main factor regulating both (16). As research progressed, the evolution of TME was found to complicate tumor formation, metastasis, and treatment (17).

**Figure 2:**
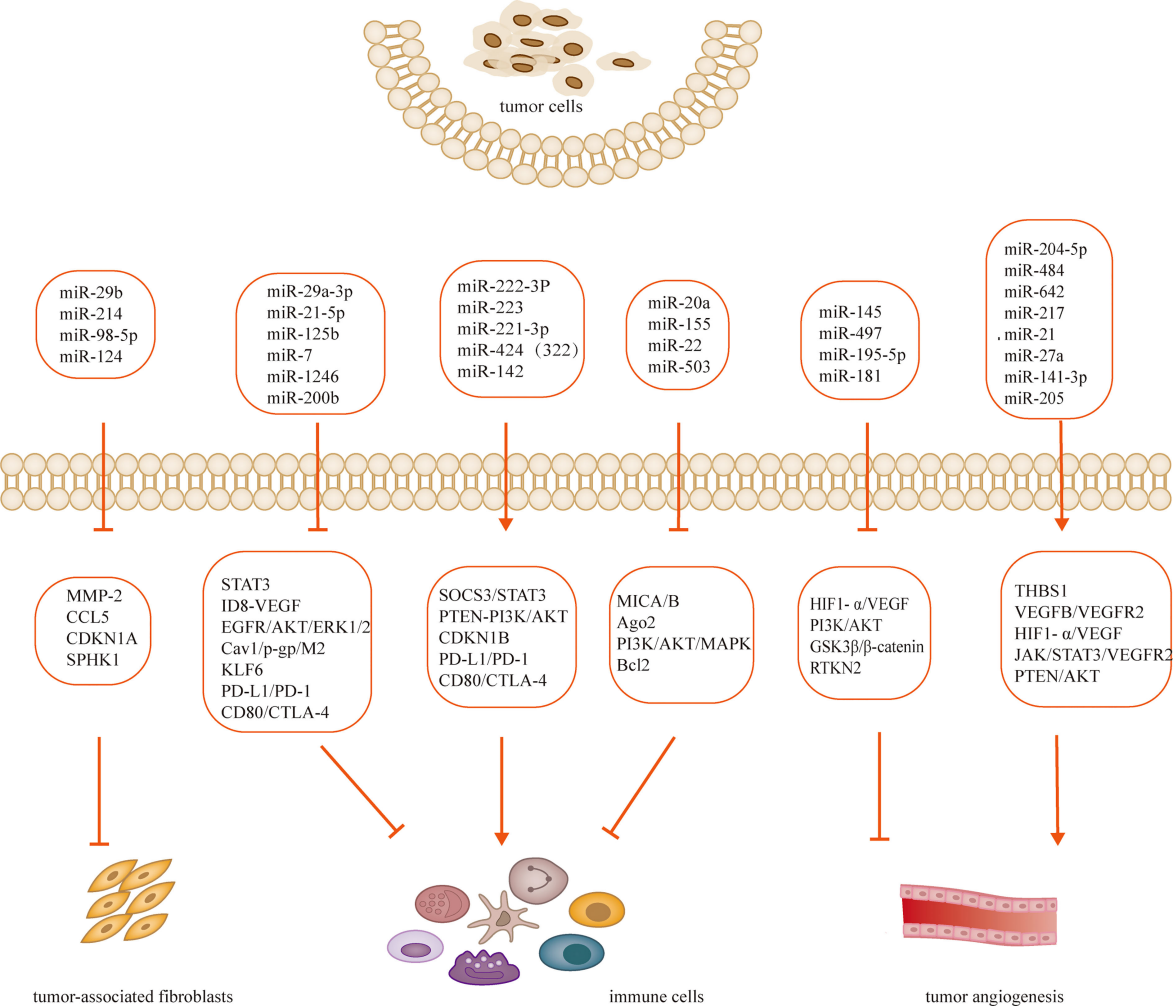
MiRNAs regulates TME and participates in the development of ovarian cancer.

Tumorigenesis, growth and metastasis are closely related to the internal and external environment in which tumor cells live, and tumor cells and their environment are both interdependent and competitive (18, 19). TME includes not only the structure, function and metabolism of tumor tissues, but also the intrinsic environment of tumor cells (20, 21). TME is complex and constantly evolving, including innate and adaptive immune cells in addition to stromal cells, fibroblasts and endothelial cells. Ovarian cancer is gynecologic cancer with high mortality rate (22), and due to the lack of characteristic clinical manifestations and effective diagnosis in the early stages, most patients have advanced disease and metastasis at the time of diagnosis. Ovarian cancer has a poor prognosis with a 5-year survival rate of approximately 47% (23). Previous studies have shown that the progression of ovarian cancer is not only associated with tumor cells but also with TME (24, 25). MiRNAs have been recognized as biomarkers for several human cancers, including ovarian cancer, and dysregulated miRNA expression is a prominent feature of ovarian cancer (26). Many studies have evaluated the expression profiles of miRNAs in tissue and serum samples from ovarian cancer patients in search of biomarkers (27–29). Several experiments have also demonstrated that miRNAs exert oncogenic or carcinogenic effects by degrading or inhibiting the translation of target mRNAs, such as miR-135a-3p (30), miR-200c (31), miR-216a (32)和miR-340 (33), these miRNAs regulate epithelial-mesenchymal transition and thus regulate the invasiveness of ovarian cancer cells. Recent studies have shown that the roles of miRNAs in TME include regulation of tumor angiogenesis (34, 35), tumor immune invasion (36, 37) and tumor interstitial interactions (12, 38), etc. (**Table 1**; **Figure 2**). In this review, we focus on the mechanisms by which miRNA-mediated regulation of TME affects the development of ovarian cancer.

**Table 1:** Detailed information of miRNAs targeting TME to regulate ovarian cancer.

miRNA	Target genes	Related hallmark	Expression	reference
miR-204-5p	THBS1	Tumor angiogenesis	Promote	(10, 39)
miR-484 miR-642 miR-217	VEGFB/VEGFR2	Tumor angiogenesis	Promote	(40)
miR-21 miR-27a	HIF1- α/VEGF	Tumor angiogenesis	Promote	(41, 42)
miR-141-3p	JAK/STAT3/VEGFR2	Tumor angiogenesis	Promote	(43)
miR-205	PTEN/AKT	Tumor angiogenesis	Promote	(44)
miR-145	HIF1- α/VEGF	Tumor angiogenesis	Inhibit	(45)
miR-497	PI3K/AKT	Tumor angiogenesis	Inhibit	(46)
miR-195-5p	GSK3β/β-catenin	Tumor angiogenesis	Inhibit	(47)
miR-181	RTKN2	Tumor angiogenesis	Inhibit	(48)
miR-29b	MMP-2	Tumor-associated fibroblast	Inhibit	(49)
miR-214	CCL5	Tumor-associated fibroblast	Inhibit	(50)
miR-98-5p	CDKN1A	Tumor-associated fibroblast	Inhibit	(51)
miR-124	SPHK1	Tumor-associated fibroblast	Inhibit	(52)
miR-29a-3p miR-21-5p	STAT3	Immune-suppressive	Inhibit	(53)
miR-125b	ID8-VEGF	Immune-suppressive	Inhibit	(54)
miR-222-3P	SOCS3/STAT3	Immune-suppressive	Promote	(55)
miR-223	PTEN-PI3K/AKT	Immune-suppressive	Promote	(56)
miR-7	EGFR/AKT/ERK1/2	Immune-suppressive	Inhibit	(57)
miR-221-3p	CDKN1B	Immune-suppressive	Promote	(58)
miR-1246	Cav1/p-gp/M2	Immune-suppressive	Inhibit	(59)
miR-200b	KLF6	Immune-suppressive	Inhibit	(60)
miR-424(322)	PD-L1/PD-1 CD80/CTLA-4	Immune-suppressive	Promote	(61)
miR-142	Sirt1	Immune activity	Promote	(62)
miR-20a	MICA/B	Immune activity	Inhibit	(63)
miR-155	Ago2	Immune activity	Inhibit	(64)
miR-22 miR-503	PI3K/AKT/MAPK Bcl2	Immune activity Immune activity	Inhibit Inhibit	(65) (65)

## MiRNAs regulate tumor angiogenesis in TME of ovarian cancer

Tumor angiogenesis is a hallmark of tumor growth, infiltration, and metastasis, and an increasing number of studies have shown its close association with TME (66, 67). Tumor growth and metastasis are dependent on the growth of blood vessels within the tumor, a process stimulated by soluble factors, of which vascular endothelial growth factor and its receptors are the main drivers (68). Recent *in vitro* and *in vivo* experiments have shown that miR-204-5p promotes ovarian tumor angiogenesis through THBS1 (39). By binding to scavenger receptor class B type 1 (SCARB1), recombinant high-density lipoprotein-nanoparticles (rHDL NPs) effectively deliver miR-204-5p inhibitors to tumors to inhibit tumor growth. This result provides new insights into miR-204-5p regulating tumor angiogenesis (39, 69). Angiogenesis plays a key role in the progression and peritoneal dissemination of ovarian cancer (70), and increased expression of VEGF has been found to promote the production of malignant ascites (71). Tumor samples from 198 ovarian cancer patients were analyzed by array and RT-PCR to confirm that three miRNAs (miR-484, miR-642 and miR-217) were able to predict chemotherapy resistance in ovarian cancer. This process is regulated by modulation of the tumor vascular system induced by the VEGFB and VEGFR2 pathways and is involved in tumor angiogenesis (40). MiR-21 and miR-27a induce ovarian cancer angiogenesis through upregulation of HIF1- α and VEGF (41, 42). Other pathways affected by miRNA dysregulation also contribute to angiogenesis in ovarian cancer. For example, miR-141-3p-containing extracellular vesicles from epithelial ovarian cancer cells promote vascular endothelial cell generation by activating the JAK/STAT3 signaling pathway and inducing VEGFR2 expression (43). Through different mechanisms, upregulation of miR-205 in ovarian cancer leads to increased angiogenesis through downregulation of the tumor suppressor PTEN and upregulation of the AKT signaling pathway (44). MiR-145 has tumor suppressive effects, and downregulation of miR-145 in ovarian cancer promotes angiogenesis through the upregulation of HIF-1 α and VEGF (45). MiR-497 targets vascular endothelial growth factor A through PI3K/AKT and MAPK/ERK pathways to inhibit ovarian cancer angiogenesis (46) Overexpression of microRNA-195-5p reduces cisplatin resistance and angiogenesis in ovarian cancer by inhibiting the psat1-dependent GSK3β/β-catenin signaling pathway (47). However, the role of aberrant regulation of miRNAs in ovarian cancer angiogenesis and development remains to be further investigated, which provides future therapeutic options and targets (72). Significant advances have been made in exploring the regulatory role of miRNAs in tumor angiogenesis. The rapidly increasing discoveries shall pave the way in the use of miRNAs as predictive biomarkers for anti-angiogenic treatments and as miRNA-based strategy against tumor angiogenesis in the future, though there are some challenges.

## MiRNAs regulate CAFs in TME of ovarian cancer

Fibroblasts are the main cells in solid tumors and are stimulated to become cancer-associated fibroblasts (CAFs) by a variety of factors secreted by tumor cells or immune cells. Activated fibroblasts gain the ability to provide fertile soil for tumor progression (73, 74). CAFs are the major tumor mesenchymal component of TME (75), promoting tumor growth, angiogenesis, invasion and metastasis through extracellular matrix, chemokines, growth factors, cytokines, and stromal degrading enzymes, and mediating drug resistance (76). Studies have shown that CAFs influence the malignant progression, metastasis, drug resistance, and recurrence of ovarian cancer. After co-culture of SKOV-3 cancer cells with primary cultured human normal fibroblasts FP-96, the expression of the tumor suppressor miR-29b was downregulated, migration of SKOV-3 cells was increased, and the activity of the miR-29b target MMP-2 was also increased (49). *In vitro* and *in vivo* experiments revealed that transient interference of three miRNAs, miR-31, miR-214 and miR-155, was sufficient to convert normal ovarian fibroblasts into induced CAFs, thereby promoting ovarian tumor growth and increasing the aggressiveness and migration of tumor cells. In contrast, the converse of this conclusion also holds, that by overexpressing downregulated miRNAs, CAFs can be reversed to more normal fibroblasts (77). Mitra et al. (50) identified one target of miR-214 as CCL5 and demonstrated that miR-214 inversely regulates CCL5. Importantly, downregulation of miR-214 increases the production of CCL5, leading to accelerated tumor growth. Anti-CCL5 antibodies blocked the effect of CAFs on tumor growth and migration. Cisplatin resistance is a common phenomenon in cancer treatment. CDKN1A was highly expressed in cisplatin-sensitive ovarian cancer cell lines, and silencing CDKN1A significantly promoted the proliferation and entry into the cell cycle of cisplatin-sensitive ovarian cancer cells and reduced apoptosis. MiR-98-5p is an exosomal miRNA derived from CAFs and promotes cisplatin resistance in ovarian cancer cells by targeting CDKN1A to inhibit CDKN1A expression (51). After miR-124 downregulation, normal fibroblasts exhibited tumor-associated fibroblast characteristics, including overexpression of α-smooth muscle actin (α-SMA) and fibroblast activated protein (FAP) and enhanced migratory and invasive abilities. Overexpression of miR-124 in CAFs reverses these features in normal fibroblasts (52). MicroRNA dysregulation is involved in the entire process of CAFs formation and executive function, and is closely related to the activation and formation of CAFs. These findings provide new insights into the communication between CAFs and cancer cells.

## MiRNAs regulate immunosuppressive cells in TME of ovarian cancer

TME is composed of many non-tumor cells called stromal cells, including tumor-associated macrophages (TAMs) (78), CAFs (79), regulatory T cells (80), myeloid-derived suppressor cells (81), endothelial cells, pericytes, and platelets (82, 83). Macrophages are the main inflammatory cells (84) and when they are present in the TME, they are called TAMs (85). Over the past decade, convincing evidence has emerged for the tumor-promoting role of macrophages in TME (20, 86, 87). TAMs are transformed from macrophages affected by cytokines, growth factors and chemokines in TME and are classified as M1 and M2 types. The M1 type has antitumor effects, whereas the M2 type has a tumor-promoting effect (78, 88). TAMs are enriched in ovarian cancer tissues and ascites and affect ovarian carcinogenesis, metastasis and invasion *via* multiple mechanisms (89, 90). It was demonstrated that miR-29a-3p and miR-21-5p synergistically inhibit STAT3, regulate Treg/Th17 cells and induce an imbalance, creating an immunosuppressive microenvironment that promotes ovarian cancer progression and metastasis (53). Hyaluronic acid nanoparticles encapsulated with miR-125b specifically target TAMs in the peritoneal cavity of ID8-VEGF ovarian cancer mice and repolarize macrophages to an immune-activating phenotype (54). It was found that miR-222-3p is enriched in epithelial ovarian cancer-derived exosomes, activates macrophage polarization toward TAMs of the M2 phenotype, and participates in the SOCS3/STAT3 pathway to promote cancer progression (55). Hypoxia triggers macrophage aggregation and induces macrophages to develop a tumor-associated macrophage-like phenotype. Exosomes released from hypoxic macrophages are enriched with miR-223, which promotes drug resistance in ovarian cancer cells *in vivo* and *in vitro via* the PTEN-PI3K/AKT pathway (56).

Ovarian cancer is prone to peritoneal metastases compared to other tumors in the abdominal cavity (91, 92). Therefore, the immune microenvironment in the peritoneum is crucial for the progression of ovarian cancer (93). Previous reports have shown that the main immune cells in the peritoneum are M2 macrophages, especially TAMs (94, 95). Microarray analysis of exosomes showed that miR-221-3p was abundant in M2 exosomes and directly inhibited cell cycle protein-dependent kinase inhibitor 1B (CDKN1B). Further, miR-221-3p promoted proliferation and G1/S transition in ovarian cancer cells (58). Cav1 is a direct target gene of miR-1246 and has been shown to be involved in exosome transfer along with multiple drug resistance genes. When ovarian cancer cells were co-cultured with macrophages, miR-1246 was able to transfer macrophages to the M2 type (59). It has been noted that miR-200b is highly expressed in plasma-derived exosomes of ovarian cancer patients and induces macrophage M2 polarization through inhibition of KLF6 expression, promoting proliferation and invasion of ovarian cancer cells (60). Accumulating literature points to the central role that many miRNAs play in the regulation of these mechanisms of macrophages-mediated immunosuppression. However, the area of research remains largely unexplored.

## MiRNAs regulate immunoreactive cells in TME of ovarian cancer

T lymphocytes are mainly divided into two subsets, CD4+ T cells and CD8+ T cells (96), and the specific immune responses they mediate are an important part of anti-tumor cellular immunity and are closely related to tumor development and prognosis (97). It was found that infiltration of CD8+ T cells was associated with prolongation of survival in tumor patients, but the inherent low immunogenicity of tumor cells with TME suppressed the immune activity of T lymphocytes, leading to a decrease in the anti-tumor capacity of T lymphocytes (98, 99) MiR-424 (322) regulates the PD-L1/PD-1 and CD80/CTLA-4 pathways in drug-resistant ovarian cancer (100), and restoration of its expression reverses the chemoresistance that accompanies PD-L1 immune checkpoint blockage (101). The synergistic effect of chemotherapy and immunotherapy is associated with the proliferation of functional cytotoxic CD8+ T cells and the suppression of bone marrow-derived suppressor cells and regulatory T cells (61). Chen et al. found that artesunate promoted apoptosis of ovarian cancer cells by promoting CD4+ T cell differentiation to Th1 through miR-142 downregulation of Sirt1 (62). It was found that miR-20a binds directly to the 3 -untranslated region of MICA/B mRNA, leading to its degradation and reducing its protein level at the plasma membrane. A reduction in membrane-bound MICA/B protein, a ligand for the natural killer group 2 member D (NKG2D) receptor found on natural killer (NK) cells, γδ+ T cells and CD8+ T cells, allows tumor cells to evade immune-mediated killing. *In vitro* and *in vivo* tumor models, antagonism of miR-20a enhanced NKG2D-mediated tumor cell killing (63).

Dendritic cells (DCs) are a specialized group of antigen-presenting cells that are the focus of initiating and regulating innate and adaptive immune responses. DCs are important in anti-tumor immunity by regulating TME, recruiting and activating anti-tumor T cells (102). An increase in the density of DCs within the TME was found to correlate with improved prognosis in cancer patients (103), yet ovarian cancer cells and TME evade immune control by impairing the activation, maturation, antigen presentation, differentiation, and recruitment of DCs (104). Min et al. demonstrated that miR-22 targets YWHAZ and blocks PI3K/Akt and MAPK signaling pathways, and miR-503 downregulates Bcl2 expression. The increased expression of miR-22 and miR-503 in tumor-associated DCs results in their reduced survival and lifespan. Thus, tumor-associated miRNAs can target a variety of intracellular signaling molecules and cause apoptosis of DCs in TME (65).

## Exosome-derived miRNAs regulate TME of ovarian cancer

**Figure 3:**
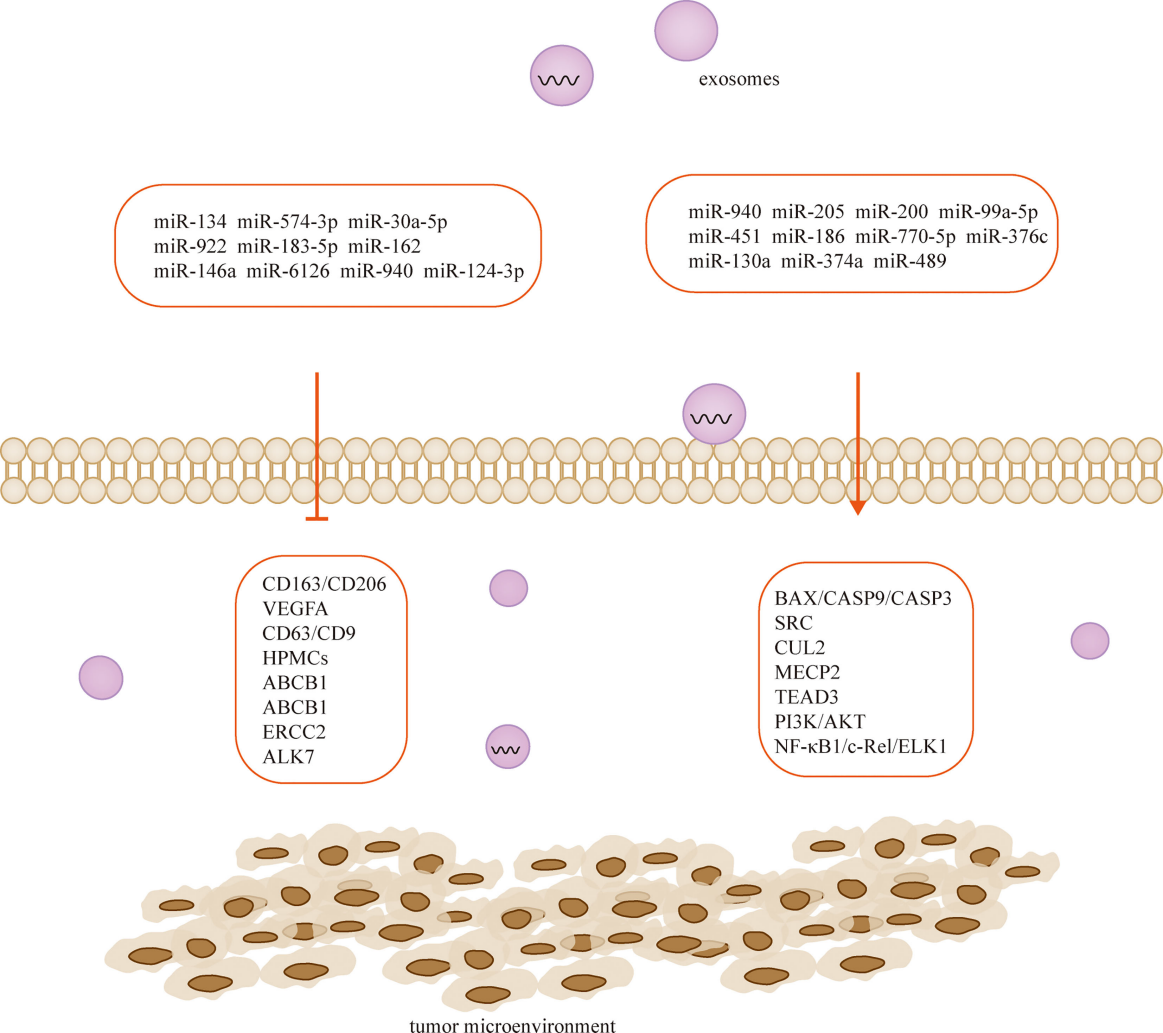
Exosome-derived miRNAs regulate TME and participate in the development of ovarian cancer.

Exosomes are tiny vesicles 30-150 nm in diameter secreted by cells, which are rich in various components such as proteins, lipids and nucleic acids and are significant in cellular communication, immune response, angiogenesis and tumorigenesis (105). There are a large number of miRNAs in exosomes (106), and exosome-derived miRNAs influence cancer progression, and they mediate ovarian cancer growth, invasion, metastasis, angiogenesis, and drug resistance through regulation of TME. Therefore, they are of great value in the early diagnosis and determination of prognosis of ovarian cancer (106, 107) (**Table 2**; **Figure 3**). MiRNAs play a role in communication between tumor cells and TME through exosome secretion and transfer (107, 124). Meanwhile, exosomal miRNA expression is dysregulated in ovarian cancer, which reflects the malignant character of the tumor to some extent (125).

**Table 2:** Details of exosome-derived miRNAs targeting the TME to regulate ovarian cancer.

miRNA	Target genes	Related hallmark	Expression	reference
miR-940	CD163/CD206	Proliferation/Migtation	Promote	(108)
miR-124-3p	BAX/CASP9/CASP3	Proliferation	Inhibit	(109)
miR-205	VEGFA	Proliferation/Migtation	Promote	(110)
miR-6126	Integrin-β1	Proliferation	Inhibit	(111)
miR-940	SRC	Proliferation	Inhibit	(112)
miR-200	CD63/CD9	Migtation	Promote	(113)
miR-99a-5p	HPMCs	Migtation	Promote	(114)
miR-574-3p miR-30a-5p miR-922	CUL2	Enhance chemosensitivity	Inhibit	(115)
miR-183-5p	MECP2	Proliferation	Inhibit	(115)
miR-162	TEAD3	Enhance chemosensitivity	Inhibit	(115)
miR-146a	PI3K/AKT	Enhance chemosensitivity	Inhibit	(116)
miR-451	ABCB1	Enhance chemosensitivity	Promote	(117)
miR-186	ABCB1	Enhance chemosensitivity	Promote	(118)
miR-770-5p	ERCC2	Enhance chemosensitivity	Promote	(119)
miR-376c	ALK7	Enhance chemosensitivity	Promote	(120)
miR-130a miR-374a	MDR1/PTEN	Enhance chemosensitivity	Promote	(121)
miR-489	AKT3	Enhance chemosensitivity	Promote	(122)
miR-134	NF-κB1/c-Rel/ELK1	Enhance chemosensitivity	Inhibit	(123)

Cancer-derived exosomal miRNAs are considered to be mediators between cancer cells and TME (126, 127). In the context of proliferation, ovarian cancer cells release exosomal miR-205 that promotes cell proliferation and invasion by targeting vascular endothelial growth factor A (128). In contrast, the widely released exosomal miR-6126 (129) and miR-940 (110) from drug-resistant and sensitive ovarian cancer cells inhibited tumor growth by targeting integrin-β1 and proto-oncogene tyrosine-protein kinase (SRC), respectively. On the other hand, non-ovarian cancer-derived exosomes also inhibit the proliferation of ovarian cancer cells (111). For example, human adipocyte-derived exosomes have inhibitory effects on two ovarian cancer cells, A2780 and SKOV-3, by blocking the cell cycle and activating the mitochondria-mediated apoptotic signaling pathway, capable of inhibiting their proliferation and wound repair (112). MiR-205 is involved in the proliferation, migration, invasion and apoptosis of ovarian cancer cells by regulating the target gene VEGFA. Transient introduction of miR-205 mimics into SKOV3 cell-derived exosomes resulted in enhanced ovarian cancer cell proliferation, migration and invasion, attenuated ovarian cancer cell apoptosis, downregulation of epithelial-mesenchymal transition protein E-cadherin, and elevated Vimentin (128). The let-7 family miRNA transcripts were found in both ovarian cancer cell lines and their exosomes and were more abundant in OVCAR-3 cells than in SKOV-3 cells. The let-7 and miR-200 families in exosomes are associated with the aggressiveness of ovarian cancer cells (130). Exosomal miR-99a-5p in the serum of ovarian cancer patients promotes invasion by increasing the expression of fibronectin and vitreous junction protein in adjacent peritoneal mesothelial cells (109). Studies have confirmed that miR-940 is highly expressed in exosomes isolated from ascites of ovarian cancer patients. Furthermore, miR-940 stimulated M2 phenotypic polarization, which in turn promoted proliferation and migration of ovarian cancer (113).

Chemotherapy is the mainstay of cancer treatment, but some patients develop chemotherapy resistance, with ovarian cancer having the highest recurrence rate associated with drug resistance, this phenomenon significantly limits the long-term outcomes of cancer patients, resulting in 5-year survival rates as low as 30%. Cellular resistance develops through long treatment cycles or intrinsic pathways. CDKN1A was highly expressed in cisplatin-sensitive ovarian cancer cell lines, and silencing CDKN1A significantly promoted the proliferation and entry into the cell cycle of cisplatin-sensitive ovarian cancer cells and reduced apoptosis. The cancer-associated fibroblast-derived exosome miR-98-5p increases ovarian cancer cell proliferation and promotes cisplatin resistance by targeting CDKN1A (51). Microarray data downloaded from the Gene Expression Omnibus database revealed that miR-574-3p, miR-30a-5p and miR-922 may mediate the HIF 1 cancer signaling pathway through regulation of CUL2, and miR-183-5p may affect cell proliferation through regulation of MECP2. Downregulation of miR-162 may promote TEAD3 expression through the Hippo signaling pathway, and this miRNA is associated with poor prognosis. Through experimental validation, researchers predicted that these genes may be potential therapeutic strategies for ovarian cancer (114). Similarly, exosomal miR-146a derived from human umbilical cord MSCs increased the sensitivity of SKOV3 ovarian cancer cells to docetaxel and paclitaxel *via* the LAMC2-mediated PI3K/Akt axis (108). Human ovarian cancer cell lines OVCAR3, A2780, A2780/DDP and A2780/Taxol were exposed to paclitaxel or cisplatin transfected with or without miR-186, and miR-186 was found to induce sensitivity of ovarian cancer cells to paclitaxel and cisplatin by targeting ABCB1. This finding demonstrates for the first time that miR-186 increases the sensitivity of ovarian cancer cells to paclitaxel and cisplatin by targeting ABCB1 and regulating the expression of GST-π (115). MiR-770-5p was significantly reduced in cisplatin-resistant patients, and it acts as an anti-oncogene that increases chemosensitivity in ovarian cancer patients by downregulating ERCC2. Thus miR-770-5p may be a useful biomarker for predicting sensitivity to cisplatin chemotherapy in ovarian cancer patients (116, 118). Activin receptor-like kinase 7 (ALK7) and its ligand Nodal induce apoptosis in human epithelial ovarian cancer cells. Ye et al. examined the regulation of ALK7 by miRNA and demonstrated that miR-376c was able to target ALK7. Overexpression of miR-376c blocked cisplatin-induced cell death, while anti-miR-376c enhanced the effect of cisplatin (119).

## Discussion

The past research that suggested that cancer develops only from changes in tumor cells has been replaced by the fact that the cellular microenvironment plays a key role in these processes. Therefore, new studies are needed to better explain the relationship between tumor cells and other cells that make up TME. Tumorigenesis and progression have causes in the tumor cells themselves as well as in TME. In recent years, miRNAs have been extensively studied, either as biomarkers or to demonstrate their potential to inhibit cellular processes. Because of this, miRNAs have great potential for the development of new cancer therapies. It was found that miRNAs are widely involved in various biological processes, including their regulatory roles in ovarian cancer progression. Several studies have demonstrated the involvement of miRNAs in the interaction between TME and ovarian cancer cells. The specific mechanisms of miRNAs are still being explored, but some miRNAs have been considered as biomarkers of tumors and have become therapeutic targets. For example, miR-204-5p, miR-484 and miR-21 promote ovarian cancer progression by regulating tumor angiogenesis in TME; miR-29b and miR-214 inhibit ovarian cancer progression by regulating CAFs; miR-125b, miR-1246 and miR-221-3p are able to inhibit/promote ovarian cancer progression by regulating immunosuppression and immunoreactive cells. These miRNAs serve as regulatory factors not only as clinical biomarkers, but also as potential therapeutic targets. It has been widely recognized that exosomes are rich in miRNAs and that exosomal miRNAs are significant in TME as signaling molecules for intercellular communication. Tumor cells transmit exosomal miRNA to cancer cells or normal cells, and conversely, fibroblasts, macrophages, etc. can also deliver exosomes to cancer cells. Exploring the role of miRNAs in TME can contribute to the search for biomarkers and probe the pathogenesis of tumors. Although many advances have been made in this area, many problems are still faced. More clinical data are needed to support the application of miRNAs as biomarkers for clinical diagnosis and detection, as well as to develop appropriate formulations for clinical treatment.

In summary, we have highlighted recent advances in the understanding of tumor microenvironmental interactions mediated by miRNAs. This article summarizes several miRNAs target important cancer cell–regulatory molecules and are involved in a complex network of signaling between cancer cells and the tumor microenvironment. In addition to their involvement in direct cell-to-cell signaling, several miRNAs are secreted through microvesicles or exosomes and affect cancer cell growth and metastasis. Some of the current challenges in miRNAs therapeutics involve selecting the right target and optimizing the delivery systems. Advances in miRNAs therapeutics have enabled us to target miRNAs alterations in a highly specific and robust manner in preclinical models. Nevertheless, studies of miRNAs-mediated interactions, specifically those focused on understanding the origin of miRNAs alterations, are needed to improve targeted therapy.

## Author contributions

All authors reviewed the literature, wrote the paper, and agree to be accountable for the work’s content. All authors contributed to the article and approved the submitted version.

## Funding

This work was supported by the National Natural Science Foundation of China (Grant No. 81973712). Jilin Province Science and Technology Development Project in China (Grant No. 20210204013YY, 20200708081YY).

## Conflict of interest

The authors declare that the research was conducted in the absence of any commercial or financial relationships that could be construed as a potential conflict of interest.

## Publisher’s note

All claims expressed in this article are solely those of the authors and do not necessarily represent those of their affiliated organizations, or those of the publisher, the editors and the reviewers. Any product that may be evaluated in this article, or claim that may be made by its manufacturer, is not guaranteed or endorsed by the publisher.

## Abbreviations

microRNA, miRNA; tumor microenvironment, TME; scavenger receptor class B type 1, SCARB1; recombinant high-density lipoprotein-nanoparticles, rHDL NPs; α-smooth muscle actin, α-SMA; fibroblast activated protein, FAP; tumor-associated macrophages, TAMs; cancer-associated fibroblasts, CAFs; cell cycle protein-dependent kinase inhibitor 1B, CDKN1B; natural killer group 2 member D, NKG2D; natural killer, NK; Dendritic cells, DCs; Activin receptor-like kinase 7, ALK7.

## References

[1] Chen P Zeng M Zhao Y Fang X . Upregulation of Limk1 caused by microRNA-138 loss aggravates the metastasis of ovarian cancer by activation of Limk1/cofilin signaling. Oncol Rep (2014) 32(5):2070–6. doi: 10.3892/or.2014.3461 25190487

[2] Sun B Liu C Li H Zhang L Luo G Liang S . Research progress on the interactions between long non-coding RNAs and microRNAs in human cancer. Oncol Lett (2020) 19(1):595–605. doi: 10.3892/ol.2019.11182 31897175 PMC6923957

[3] Konishi H Sato H Takahashi K Fujiya M . Tumor-progressive mechanisms mediating miRNA-protein interaction. Int J Mol Sci (2021) 22(22):12303. doi: 10.3390/ijms222212303 34830186 PMC8622902

[4] Xu Y Wu W Han Q Wang Y Li C Zhang P . New insights into the interplay between non-coding RNAs and RNA-binding protein HnRNPK in regulating cellular functions. Cells (2019) 8(1):62. doi: 10.3390/cells8010062 30658384 PMC6357021

[5] Sánchez-Sendra B García-Giménez JL González-Muñoz JF Navarro L Murgui A Terrádez L . Circulating miRNA expression analysis reveals new potential biomarkers for human cutaneous melanoma staging. J Eur Acad Dermatol Venereol (2020) 34(3):e126–e9. doi: 10.1111/jdv.16060 31710393

[6] Sabry D El-Deek SEM Maher M El-Baz MAH El-Bader HM Amer E . Role of miRNA-210, miRNA-21 and miRNA-126 as diagnostic biomarkers in colorectal carcinoma: impact of HIF-1α-VEGF signaling pathway. Mol Cell Biochem (2019) 454(1-2):177–89. doi: 10.1007/s11010-018-3462-1 30357530

[7] Condrat CE Thompson DC Barbu MG Bugnar OL Boboc A Cretoiu D . miRNAs as biomarkers in disease: Latest findings regarding their role in diagnosis and prognosis. Cells (2020) 9(2):276. doi: 10.3390/cells9020276 31979244 PMC7072450

[8] Annese T Tamma R De Giorgis M Ribatti D . microRNAs biogenesis, functions and role in tumor angiogenesis. Front Oncol (2020) 10:581007. doi: 10.3389/fonc.2020.581007 33330058 PMC7729128

[9] Leone P Buonavoglia A Fasano R Solimando AG De Re V Cicco S . Insights into the regulation of tumor angiogenesis by micro-RNAs. J Clin Med (2019) 8(12):2030. doi: 10.3390/jcm8122030 31757094 PMC6947031

[10] Yi M Xu L Jiao Y Luo S Li A Wu K . The role of cancer-derived microRNAs in cancer immune escape. J Hematol Oncol (2020) 13(1):25. doi: 10.1186/s13045-020-00848-8 32222150 PMC7103070

[11] Zheng J Yang T Gao S Cheng M Shao Y Xi Y . miR-148a-3p silences the CANX/MHC-I pathway and impairs CD8(+) T cell-mediated immune attack in colorectal cancer. FASEB J (2021) 35(8):e21776. doi: 10.1096/fj.202100235R 34324740

[12] Terkelsen T Russo F Gromov P Haakensen VD Brunak S Gromova I . Secreted breast tumor interstitial fluid microRNAs and their target genes are associated with triple-negative breast cancer, tumor grade, and immune infiltration. Breast Cancer Res (2020) 22(1):73. doi: 10.1186/s13058-020-01295-6 32605588 PMC7329449

[13] da Cunha BR Domingos C Stefanini ACB Henrique T Polachini GM Castelo-Branco P . Cellular interactions in the tumor microenvironment: The role of secretome. J Cancer (2019) 10(19):4574–87. doi: 10.7150/jca.21780 PMC6746126 31528221

[14] Koliaraki V Prados A Armaka M Kollias G . The mesenchymal context in inflammation, immunity and cancer. Nat Immunol (2020) 21(9):974–82. doi: 10.1038/s41590-020-0741-2 32747813

[15] Kobayashi H Enomoto A Woods SL Burt AD Takahashi M Worthley DL . Cancer-associated fibroblasts in gastrointestinal cancer. Nat Rev Gastroenterol Hepatol (2019) 16(5):282–95. doi: 10.1038/s41575-019-0115-0 30778141

[16] Neophytou CM Panagi M Stylianopoulos T Papageorgis P . The role of tumor microenvironment in cancer metastasis: Molecular mechanisms and therapeutic opportunities. Cancers (2021) 13(9):2053. doi: 10.3390/cancers13092053 33922795 PMC8122975

[17] Zhuyan J Chen M Zhu T Bao X Zhen T Xing K . Critical steps to tumor metastasis: alterations of tumor microenvironment and extracellular matrix in the formation of pre-metastatic and metastatic niche. Cell biosci (2020) 10:89. doi: 10.1186/s13578-020-00453-9 32742634 PMC7388444

[18] Paduch R . The role of lymphangiogenesis and angiogenesis in tumor metastasis. Cell Oncol (Dordrecht) (2016) 39(5):397–410. doi: 10.1007/s13402-016-0281-9 PMC5052283 27126599

[19] Sau S Alsaab HO Bhise K Alzhrani R Nabil G Iyer AK . Multifunctional nanoparticles for cancer immunotherapy: A groundbreaking approach for reprogramming malfunctioned tumor environment. J Controlled release (2018) 274:24–34. doi: 10.1016/j.jconrel.2018.01.028 PMC5847475 29391232

[20] Wang H Yung MMH Ngan HYS Chan KKL Chan DW . The impact of the tumor microenvironment on macrophage polarization in cancer metastatic progression. Int J Mol Sci (2021) 22(12):6560. doi: 10.3390/ijms22126560 34207286 PMC8235734

[21] Renner K Singer K Koehl GE Geissler EK Peter K Siska PJ . Metabolic hallmarks of tumor and immune cells in the tumor microenvironment. Front Immunol (2017) 8:248. doi: 10.3389/fimmu.2017.00248 28337200 PMC5340776

[22] Ha HI Chang HK Park SJ Lim J Won YJ Lim MC . The incidence and survival of cervical, ovarian, and endometrial cancer in Korea, 1999-2017: Korea central cancer registry. Obstetrics gynecol Sci (2021) 64(5):444–53. doi: 10.5468/ogs.21116 PMC8458610 34399564

[23] Moufarrij S Dandapani M Arthofer E Gomez S Srivastava A Lopez-Acevedo M . Epigenetic therapy for ovarian cancer: promise and progress. Clin Epigenet (2019) 11(1):7. doi: 10.1186/s13148-018-0602-0 PMC6334391 30646939

[24] Shi R Tang YQ Miao H . Metabolism in tumor microenvironment: Implications for cancer immunotherapy. MedComm (2020) 1(1):47–68. doi: 10.1002/mco2.6 34766109 PMC8489668

[25] Guan X Zong ZH Liu Y Chen S Wang LL Zhao Y . circPUM1 promotes tumorigenesis and progression of ovarian cancer by sponging miR-615-5p and miR-6753-5p. Mol Ther Nucleic Acids (2019) 18:882–92. doi: 10.1016/j.omtn.2019.09.032 PMC6881671 31751911

[26] Staicu CE Predescu DV Rusu CM Radu BM Cretoiu D Suciu N . Role of microRNAs as clinical cancer biomarkers for ovarian cancer: A short overview. Cells (2020) 9(1):169. doi: 10.3390/cells9010169 31936634 PMC7016727

[27] Taylor DD Gercel-Taylor C . MicroRNA signatures of tumor-derived exosomes as diagnostic biomarkers of ovarian cancer. Gynecol Oncol (2008) 110(1):13–21. doi: 10.1016/j.ygyno.2008.04.033 18589210

[28] Resnick KE Alder H Hagan JP Richardson DL Croce CM Cohn DE . The detection of differentially expressed microRNAs from the serum of ovarian cancer patients using a novel real-time PCR platform. Gynecol Oncol (2009) 112(1):55–9. doi: 10.1016/j.ygyno.2008.08.036 18954897

[29] Wang W Yin Y Shan X Zhou X Liu P Cao Q . The value of plasma-based MicroRNAs as diagnostic biomarkers for ovarian cancer. Am J Med Sci (2019) 358(4):256–67. doi: 10.1016/j.amjms.2019.07.005 31353030

[30] Duan S Dong X Hai J Jiang J Wang W Yang J . MicroRNA-135a-3p is downregulated and serves as a tumour suppressor in ovarian cancer by targeting CCR2. Biomed pharmacother = Biomed pharmacother (2018) 107:712–20. doi: 10.1016/j.biopha.2018.08.044 30138893

[31] Koutsaki M Spandidos DA Zaravinos A . Epithelial-mesenchymal transition-associated miRNAs in ovarian carcinoma, with highlight on the miR-200 family: prognostic value and prospective role in ovarian cancer therapeutics. Cancer Lett (2014) 351(2):173–81. doi: 10.1016/j.canlet.2014.05.022 24952258

[32] Ghafouri-Fard S Shoorei H Taheri M . miRNA profile in ovarian cancer. Exp Mol Pathol (2020) 113:104381. doi: 10.1016/j.yexmp.2020.104381 31954715

[33] Huang Z Xu Y Wan M Zeng X Wu J . miR-340: A multifunctional role in human malignant diseases. Int J Biol Sci (2021) 17(1):236–46. doi: 10.7150/ijbs.51123 PMC7757049 33390846

[34] Wang Y Wang L Chen C Chu X . New insights into the regulatory role of microRNA in tumor angiogenesis and clinical implications. Mol Cancer (2018) 17(1):22. doi: 10.1186/s12943-018-0766-4 29415727 PMC5804051

[35] Goradel NH Mohammadi N Haghi-Aminjan H Farhood B Negahdari B Sahebkar A . Regulation of tumor angiogenesis by microRNAs: State of the art. J Cell Physiol (2019) 234(2):1099–110. doi: 10.1002/jcp.27051 30070704

[36] Hong W Xue M Jiang J Zhang Y Gao X . Circular RNA circ-CPA4/ let-7 miRNA/PD-L1 axis regulates cell growth, stemness, drug resistance and immune evasion in non-small cell lung cancer (NSCLC). J Exp Clin Cancer Res (2020) 39(1):149. doi: 10.1186/s13046-020-01648-1 32746878 PMC7397626

[37] Chen C Liu JM Luo YP . MicroRNAs in tumor immunity: functional regulation in tumor-associated macrophages. J Zhejiang Univ Sci B (2020) 21(1):12–28. doi: 10.1631/jzus.B1900452 31898439 PMC6964996

[38] Wu C Zhuang Y Jiang S Liu S Zhou J Wu J . Interaction between wnt/β-catenin pathway and microRNAs regulates epithelial-mesenchymal transition in gastric cancer (Review). Int J Oncol (2016) 48(6):2236–46. doi: 10.3892/ijo.2016.3480 27082441

[39] Chen X Mangala LS Mooberry L Bayraktar E Dasari SK Ma S . Identifying and targeting angiogenesis-related microRNAs in ovarian cancer. Oncogene (2019) 38(33):6095–108. doi: 10.1038/s41388-019-0862-y PMC7293105 31289363

[40] Vecchione A Belletti B Lovat F Volinia S Chiappetta G Giglio S . A microRNA signature defines chemoresistance in ovarian cancer through modulation of angiogenesis. Proc Natl Acad Sci United States America (2013) 110(24):9845–50. doi: 10.1073/pnas.1305472110 PMC3683704 23697367

[41] Xie Z Cao L Zhang J . miR-21 modulates paclitaxel sensitivity and hypoxia-inducible factor-1α expression in human ovarian cancer cells. Oncol Lett (2013) 6(3):795–800. doi: 10.3892/ol.2013.1432 24137413 PMC3789026

[42] Lai Y Zhang X Zhang Z Shu Y Luo X Yang Y . The microRNA-27a: ZBTB10-specificity protein pathway is involved in follicle stimulating hormone-induced VEGF, Cox2 and survivin expression in ovarian epithelial cancer cells. Int J Oncol (2013) 42(2):776–84. doi: 10.3892/ijo.2012.1743 23254909

[43] Masoumi-Dehghi S Babashah S Sadeghizadeh M . microRNA-141-3p-containing small extracellular vesicles derived from epithelial ovarian cancer cells promote endothelial cell angiogenesis through activating the JAK/STAT3 and NF-κB signaling pathways. J Cell communication Signaling (2020) 14(2):233–44. doi: 10.1007/s12079-020-00548-5 PMC7272520 32034654

[44] He L Zhu W Chen Q Yuan Y Wang Y Wang J . Ovarian cancer cell-secreted exosomal miR-205 promotes metastasis by inducing angiogenesis. Theranostics (2019) 9(26):8206–20. doi: 10.7150/thno.37455 PMC6857047 31754391

[45] Xu Q Liu LZ Qian X Chen Q Jiang Y Li D . MiR-145 directly targets p70S6K1 in cancer cells to inhibit tumor growth and angiogenesis. Nucleic Acids Res (2012) 40(2):761–74. doi: 10.1093/nar/gkr730 PMC3258133 21917858

[46] Wang W Ren F Wu Q Jiang D Li H Shi H . MicroRNA-497 suppresses angiogenesis by targeting vascular endothelial growth factor a through the PI3K/AKT and MAPK/ERK pathways in ovarian cancer. Oncol Rep (2014) 32(5):2127–33. doi: 10.3892/or.2014.3439 25176450

[47] Dai J Wei R Zhang P . Retraction note: Overexpression of microRNA-195-5p reduces cisplatin resistance and angiogenesis in ovarian cancer by inhibiting the PSAT1-dependent GSK3β/β-catenin signaling pathway. J Trans Med (2022) 20(1):351. doi: 10.1186/s12967-022-03560-y PMC9354355 35927652

[48] Lin Z Li D Cheng W Wu J Wang K Hu Y . MicroRNA-181 functions as an antioncogene and mediates NF-κB pathway by targeting RTKN2 in ovarian cancers. Reprod Sci (Thousand Oaks Calif) (2019) 26(8):1071–81. doi: 10.1177/1933719118805865 30309296

[49] Medeiros M Ribeiro AO Lupi LA Romualdo GR Pinhal D Chuffa LGA . Mimicking the tumor microenvironment: Fibroblasts reduce miR-29b expression and increase the motility of ovarian cancer cells in a co-culture model. Biochem Biophys Res Commun (2019) 516(1):96–101. doi: 10.1016/j.bbrc.2019.06.001 31200958

[50] Mitra AK Zillhardt M Hua Y Tiwari P Lengyel EJCD . MicroRNAs reprogram normal fibroblasts into cancer-associated fibroblasts in ovarian cancer. Cancer discovery (2012) 2(12):1100–8. doi: 10.1158/2159-8290.cd-12-0206 PMC3685866 23171795

[51] Guo H Ha C Dong H Yang Z Ma Y Ding Y . Cancer-associated fibroblast-derived exosomal microRNA-98-5p promotes cisplatin resistance in ovarian cancer by targeting CDKN1A. Cancer Cell Int (2019) 19:347. doi: 10.1186/s12935-019-1051-3 31889899 PMC6925473

[52] Zhang Y Cai H Chen S Sun D Zhang D He Y . Exosomal transfer of miR-124 inhibits normal fibroblasts to cancer-associated fibroblasts transition by targeting sphingosine kinase 1 in ovarian cancer. J Cell Biochem (2019) 120(8):13187–201. doi: 10.1002/jcb.28593 30957275

[53] Zhou J Li X Wu X Zhang T Zhu Q Wang X . Exosomes released from tumor-associated macrophages transfer miRNAs that induce a Treg/Th17 cell imbalance in epithelial ovarian cancer. Cancer Immunol Res (2018) 6(12):1578–92. doi: 10.1158/2326-6066.CIR-17-0479 30396909

[54] Parayath NN Gandham SK Leslie F Amiji MM . Improved anti-tumor efficacy of paclitaxel in combination with MicroRNA-125b-based tumor-associated macrophage repolarization in epithelial ovarian cancer. Cancer Lett (2019) 461:1–9. doi: 10.1016/j.canlet.2019.07.002 31288064 PMC6682447

[55] Ying X Wu Q Wu X Zhu Q Wang X Jiang L . Epithelial ovarian cancer-secreted exosomal miR-222-3p induces polarization of tumor-associated macrophages. Oncotarget (2016) 7(28):43076–87. doi: 10.18632/oncotarget.9246 PMC5190009 27172798

[56] Zhu X Shen H Yin X Yang M Wei H Chen Q . Macrophages derived exosomes deliver miR-223 to epithelial ovarian cancer cells to elicit a chemoresistant phenotype. J Exp Clin Cancer Res (2019) 38(1):81. doi: 10.1186/s13046-019-1095-1 30770776 PMC6377760

[57] Hu Y Li D Wu A Qiu X Di W Huang L . TWEAK-stimulated macrophages inhibit metastasis of epithelial ovarian cancer *via* exosomal shuttling of microRNA. Cancer Lett (2017) 393:60–7. doi: 10.1016/j.canlet.2017.02.009 28216373

[58] Li X Tang M . Exosomes released from M2 macrophages transfer miR-221-3p contributed to EOC progression through targeting CDKN1B. Cancer Med (2020) 9(16):5976–88. doi: 10.1002/cam4.3252 PMC7433826 32590883

[59] Kanlikilicer P Bayraktar R Denizli M Rashed MH Ivan C Aslan B . Exosomal miRNA confers chemo resistance *via* targeting Cav1/p-gp/M2-type macrophage axis in ovarian cancer. EBioMedicine (2018) 38:100–12. doi: 10.1016/j.ebiom.2018.11.004 PMC6306310 30487062

[60] Xiong J He X Xu Y Zhang W Fu F . MiR-200b is upregulated in plasma-derived exosomes and functions as an oncogene by promoting macrophage M2 polarization in ovarian cancer. J Ovarian Res (2021) 14(1):74. doi: 10.1186/s13048-021-00826-9 34078414 PMC8170822

[61] Xu S Tao Z Hai B Liang H Shi Y Wang T . miR-424(322) reverses chemoresistance *via* T-cell immune response activation by blocking the PD-L1 immune checkpoint. Nat Commun (2016) 7:11406. doi: 10.1038/ncomms11406 27147225 PMC4858750

[62] Chen X Zhang XL Zhang GH Gao YF . Artesunate promotes Th1 differentiation from CD4+ T cells to enhance cell apoptosis in ovarian cancer *via* miR-142. Braz J Med Biol Res = Rev Bras pesquisas medicas e biologicas (2019) 52(5):e7992. doi: 10.1590/1414-431x20197992 PMC6489539 31038546

[63] Xie J Liu M Li Y Nie Y Mi Q Zhao S . Ovarian tumor-associated microRNA-20a decreases natural killer cell cytotoxicity by downregulating MICA/B expression. Cell Mol Immunol (2014) 11(5):495–502. doi: 10.1038/cmi.2014.30 24813230 PMC4197204

[64] Cubillos-Ruiz JR Baird JR Tesone AJ Rutkowski MR Scarlett UK Camposeco-Jacobs AL . Reprogramming tumor-associated dendritic cells *in vivo* using miRNA mimetics triggers protective immunity against ovarian cancer. Cancer Res (2012) 72(7):1683–93. doi: 10.1158/0008-5472.CAN-11-3160 PMC3319850 22307839

[65] Min S Liang X Zhang M Zhang Y Mei S Liu J . Multiple tumor-associated microRNAs modulate the survival and longevity of dendritic cells by targeting YWHAZ and Bcl2 signaling pathways. J Immunol (Baltimore Md 1950) (2013) 190(5):2437–46. doi: 10.4049/jimmunol.1202282 23355742

[66] Chandler KB Costello CE Rahimi N . Glycosylation in the tumor microenvironment: Implications for tumor angiogenesis and metastasis. Cells (2019) 8(6):544. doi: 10.3390/cells8060544 31195728 PMC6627046

[67] Wei X Chen Y Jiang X Peng M Liu Y Mo Y . Mechanisms of vasculogenic mimicry in hypoxic tumor microenvironments. Mol Cancer (2021) 20(1):7. doi: 10.1186/s12943-020-01288-1 33397409 PMC7784348

[68] Mariotti V Fiorotto R Cadamuro M Fabris L Strazzabosco M . New insights on the role of vascular endothelial growth factor in biliary pathophysiology. JHEP Rep Innovation Hepatol (2021) 3(3):100251. doi: 10.1016/j.jhepr.2021.100251 PMC8189933 34151244

[69] Lawler PR Lawler J . Molecular basis for the regulation of angiogenesis by thrombospondin-1 and -2. Cold Spring Harbor Perspect Med (2012) 2(5):a006627. doi: 10.1101/cshperspect.a006627 PMC3331684 22553494

[70] Dai L Song K Di W . Adipocytes: active facilitators in epithelial ovarian cancer progression? J Ovarian Res (2020) 13(1):115. doi: 10.1186/s13048-020-00718-4 32967712 PMC7513299

[71] Chen Y Mathy NW Lu H . The role of VEGF in the diagnosis and treatment of malignant pleural effusion in patients with non−small cell lung cancer (Review). Mol Med Rep (2018) 17(6):8019–30. doi: 10.3892/mmr.2018.8922 PMC5983970 29693703

[72] Stieg DC Wang Y Liu LZ Jiang BH . ROS and miRNA dysregulation in ovarian cancer development, angiogenesis and therapeutic resistance. Int J Mol Sci (2022) 23(12):6702. doi: 10.3390/ijms23126702 35743145 PMC9223852

[73] Kuzet SE Gaggioli C . Fibroblast activation in cancer: when seed fertilizes soil. Cell Tissue Res (2016) 365(3):607–19. doi: 10.1007/s00441-016-2467-x 27474009

[74] Barrett RL Puré E . Cancer-associated fibroblasts and their influence on tumor immunity and immunotherapy. eLife (2020) 9:e57243. doi: 10.7554/elife.57243 33370234 PMC7769568

[75] Kalluri R . The biology and function of fibroblasts in cancer. Nat Rev Cancer (2016) 16(9):582–98. doi: 10.1038/nrc.2016.73 27550820

[76] Cui Y Wang D Xie M . Tumor-derived extracellular vesicles promote activation of carcinoma-associated fibroblasts and facilitate invasion and metastasis of ovarian cancer by carrying miR-630. Front Cell Dev Biol (2021) 9:652322. doi: 10.3389/fcell.2021.652322 34277601 PMC8277948

[77] Chou J Werb ZJCD . MicroRNAs play a big role in regulating ovarian cancer-associated fibroblasts and the tumor microenvironment. Cancer discovery (2012) 2(12):1078–80. doi: 10.1158/2159-8290.CD-12-0465 PMC3531831 23230184

[78] Zhou K Cheng T Zhan J Peng X Zhang Y Wen J . Targeting tumor-associated macrophages in the tumor microenvironment. Oncol Lett (2020) 20(5):234. doi: 10.3892/ol.2020.12097 32968456 PMC7500051

[79] Kochetkova M Samuel MS . Differentiation of the tumor microenvironment: are CAFs the organizer? Trends Cell Biol (2022) 32(4):285–94. doi: 10.1016/j.tcb.2021.11.008 34895986

[80] Li C Jiang P Wei S Xu X Wang J . Regulatory T cells in tumor microenvironment: new mechanisms, potential therapeutic strategies and future prospects. Mol Cancer (2020) 19(1):116. doi: 10.1186/s12943-020-01234-1 32680511 PMC7367382

[81] Tian X Shen H Li Z Wang T Wang S . Tumor-derived exosomes, myeloid-derived suppressor cells, and tumor microenvironment. J Hematol Oncol (2019) 12(1):84. doi: 10.1186/s13045-019-0772-z 31438991 PMC6704713

[82] Jin J Lin J Xu A Lou J Qian C Li X . CCL2: An important mediator between tumor cells and host cells in tumor microenvironment. Front Oncol (2021) 11:722916. doi: 10.3389/fonc.2021.722916 34386431 PMC8354025

[83] Su T Zhang P Zhao F Zhang S . Exosomal MicroRNAs mediating crosstalk between cancer cells with cancer-associated fibroblasts and tumor-associated macrophages in the tumor microenvironment. Front Oncol (2021) 11:631703. doi: 10.3389/fonc.2021.631703 33869017 PMC8049566

[84] Savelli G Bonacina M Rizzo A Zaniboni A . Activated macrophages are the main inflammatory cell in COVID-19 interstitial pneumonia infiltrates. is it possible to show their metabolic activity and thus the grade of inflammatory burden with (18)F-fluorocholine PET/CT? Med Hypotheses (2020) 144:109885. doi: 10.1016/j.mehy.2020.109885 32540605 PMC7252431

[85] Malekghasemi S Majidi J Baghbanzadeh A Abdolalizadeh J Baradaran B Aghebati-Maleki L . Tumor-associated macrophages: Protumoral macrophages in inflammatory tumor microenvironment. Advanced Pharm Bull (2020) 10(4):556–65. doi: 10.34172/apb.2020.066 PMC7539304 33062602

[86] Lopez-Bergami P Barbero G . The emerging role of Wnt5a in the promotion of a pro-inflammatory and immunosuppressive tumor microenvironment. Cancer metastasis Rev (2020) 39(3):933–52. doi: 10.1007/s10555-020-09878-7 32435939

[87] Yang E Wang X Gong Z Yu M Wu H Zhang D . Exosome-mediated metabolic reprogramming: the emerging role in tumor microenvironment remodeling and its influence on cancer progression. Signal transduction targeted Ther (2020) 5(1):242. doi: 10.1038/s41392-020-00359-5 PMC7572387 33077737

[88] Wang J Li D Cang H Guo B . Crosstalk between cancer and immune cells: Role of tumor-associated macrophages in the tumor microenvironment. Cancer Med (2019) 8(10):4709–21. doi: 10.1002/cam4.2327 PMC6712467 31222971

[89] Motohara T Masuda K Morotti M Zheng Y El-Sahhar S Chong KY . An evolving story of the metastatic voyage of ovarian cancer cells: cellular and molecular orchestration of the adipose-rich metastatic microenvironment. Oncogene (2019) 38(16):2885–98. doi: 10.1038/s41388-018-0637-x PMC6755962 30568223

[90] Yousefi M Dehghani S Nosrati R Ghanei M Salmaninejad A Rajaie S . Current insights into the metastasis of epithelial ovarian cancer - hopes and hurdles. Cell Oncol (Dordrecht) (2020) 43(4):515–38. doi: 10.1007/s13402-020-00513-9 PMC12990730 32418122

[91] van Baal J van Noorden CJF Nieuwland R Van de Vijver KK Sturk A van Driel WJ . Development of peritoneal carcinomatosis in epithelial ovarian cancer: A review. J Histochem Cytochem (2018) 66(2):67–83. doi: 10.1369/0022155417742897 29164988 PMC5794203

[92] Roque R Costa Sousa F Figueiredo-Dias M . Epithelial-mesenchymal interconversions in ovarian cancer: The levels and functions of e-cadherin in intraabdominal dissemination. Oncol Rev (2020) 14(2):475. doi: 10.4081/oncol.2020.475 32676171 PMC7358986

[93] Baci D Bosi A Gallazzi M Rizzi M Noonan DM Poggi A . The ovarian cancer tumor immune microenvironment (TIME) as target for therapy: A focus on innate immunity cells as therapeutic effectors. Int J Mol Sci (2020) 21(9):3125. doi: 10.3390/ijms21093125 32354198 PMC7247443

[94] An Y Yang Q . Tumor-associated macrophage-targeted therapeutics in ovarian cancer. Int J Cancer (2021) 149(1):21–30. doi: 10.1002/ijc.33408 33231290

[95] Nowak M Klink M . The role of tumor-associated macrophages in the progression and chemoresistance of ovarian cancer. Cells (2020) 9(5):846–63. doi: 10.3390/cells9051299 PMC7290435 32456078

[96] Brown CC Gudjonson H Pritykin Y Deep D Lavallée VP Mendoza A . Transcriptional basis of mouse and human dendritic cell heterogeneity. Cell (2019) 179(4):846–63. e24. doi: 10.1016/j.cell.2019.09.035 31668803 PMC6838684

[97] Liu Y Mikrani R Xie D Wazir J Shrestha S Ullah R . Chronic prostatitis/chronic pelvic pain syndrome and prostate cancer: study of immune cells and cytokines. Fundam Clin Pharmacol (2020) 34(2):160–72. doi: 10.1111/fcp.12517 31642541

[98] Peggs KS Quezada SA Allison JP . Cell intrinsic mechanisms of T-cell inhibition and application to cancer therapy. Immunol Rev (2008) 224:141–65. doi: 10.1111/j.1600-065X.2008.00649.x 18759925

[99] Nicholas NS Apollonio B Ramsay AG . Tumor microenvironment (TME)-driven immune suppression in b cell malignancy. Biochim Biophys Acta (2016) 1863(3):471–82. doi: 10.1016/j.bbamcr.2015.11.003 26554850

[100] Li S Wu Y Zhang J Sun H Wang X . Role of miRNA-424 in cancers. OncoTargets Ther (2020) 13:9611–22. doi: 10.2147/OTT.S266541 PMC7532073 33061443

[101] Vishnubalaji R Shaath H Elango R Alajez NM . Noncoding RNAs as potential mediators of resistance to cancer immunotherapy. Semin Cancer Biol (2020) 65:65–79. doi: 10.1016/j.semcancer.2019.11.006 31733291

[102] Liu Y Zhao JJ Zhou ZQ Pan QZ Zhu Q Tang Y . IL-37 induces anti-tumor immunity by indirectly promoting dendritic cell recruitment and activation in hepatocellular carcinoma. Cancer Manage Res (2019) 11:6691. doi: 10.2147/CMAR.S200627 PMC6646800 31410060

[103] Wculek SK Cueto FJ Mujal AM Melero I Krummel MF Sancho D . Dendritic cells in cancer immunology and immunotherapy. Nat Rev Immunol (2020) 20(1):7–24. doi: 10.1038/s41577-019-0210-z 31467405

[104] Jiang Y Wang C Zhou S . Targeting tumor microenvironment in ovarian cancer: Premise and promise. Biochim Biophys Acta Rev Cancer (2020) 1873(2):188361. doi: 10.1016/j.bbcan.2020.188361 32234508

[105] Gurung S Perocheau D Touramanidou L Baruteau J . The exosome journey: from biogenesis to uptake and intracellular signalling. Cell communication Signaling (2021) 19(1):47. doi: 10.1186/s12964-021-00730-1 33892745 PMC8063428

[106] Zhao L Liu W Xiao J Cao B . The role of exosomes and "exosomal shuttle microRNA" in tumorigenesis and drug resistance. Cancer Lett (2015) 356(2 Pt B):339–46. doi: 10.1016/j.canlet.2014.10.027 25449429

[107] Malla RR Pandrangi S Kumari S Gavara MM Badana AK . Exosomal tetraspanins as regulators of cancer progression and metastasis and novel diagnostic markers. Asia-Pacific J Clin Oncol (2018) 14(6):383–91. doi: 10.1111/ajco.12869 29575602

[108] Chen X Ying X Wang X Wu X Zhu Q Wang X . Exosomes derived from hypoxic epithelial ovarian cancer deliver microRNA-940 to induce macrophage M2 polarization. Oncol Rep (2017) 38(1):522–8. doi: 10.3892/or.2017.5697 28586039

[109] Reza A Choi YJ Yasuda H Kim JH . Human adipose mesenchymal stem cell-derived exosomal-miRNAs are critical factors for inducing anti-proliferation signalling to A2780 and SKOV-3 ovarian cancer cells. Sci Rep (2016) 6:38498. doi: 10.1038/srep38498 27929108 PMC5143979

[110] Wang L Zhao F Xiao Z Yao L . Exosomal microRNA-205 is involved in proliferation, migration, invasion, and apoptosis of ovarian cancer cells *via* regulating VEGFA. Cancer Cell Int (2019) 19:281. doi: 10.1186/s12935-019-0990-z 31719795 PMC6836480

[111] Kanlikilicer P Saber M Bayraktar R Mitra R Ivan C Aslan B . Ubiquitous release of exosomal tumor suppressor miR-6126 from ovarian cancer cells. Cancer research (2016) 76(24):7194–207. doi: 10.1158/0008-5472.CAN-16-0714 PMC5901763 27742688

[112] Rashed MH Kanlikilicer P Rodriguez-Aguayo C Pichler M Bayraktar R Bayraktar E . Exosomal miR-940 maintains SRC-mediated oncogenic activity in cancer cells: a possible role for exosomal disposal of tumor suppressor miRNAs. Oncotarget (2017) 8(12):20145–64. doi: 10.18632/oncotarget.15525 PMC5386751 28423620

[113] Kobayashi M Salomon C Tapia J Illanes SE Mitchell MD Rice GE . Ovarian cancer cell invasiveness is associated with discordant exosomal sequestration of let-7 miRNA and miR-200. J Trans Med (2014) 12:4. doi: 10.1186/1479-5876-12-4 PMC3896684 24393345

[114] Yoshimura A Sawada K Nakamura K Kinose Y Nakatsuka E Kobayashi M . Exosomal miR-99a-5p is elevated in sera of ovarian cancer patients and promotes cancer cell invasion by increasing fibronectin and vitronectin expression in neighboring peritoneal mesothelial cells. BMC Cancer (2018) 18(1):1065. doi: 10.1186/s12885-018-4974-5 30396333 PMC6217763

[115] Feng Y Hang W Sang Z Li S Xu W Miao Y . Identification of exosomal and non−exosomal microRNAs associated with the drug resistance of ovarian cancer. Mol Med Rep (2019) 19(5):3376–92. doi: 10.3892/mmr.2019.10008 PMC6471492 30864705

[116] Qiu L Wang J Chen M Chen F Tu W . Exosomal microRNA−146a derived from mesenchymal stem cells increases the sensitivity of ovarian cancer cells to docetaxel and taxane *via* a LAMC2−mediated PI3K/Akt axis. Int J Mol Med (2020) 46(2):609–20. doi: 10.3892/ijmm.2020.4634 PMC7307828 32626953

[117] Zhu H Wu H Liu X Evans BR Medina DJ Liu CG . Role of MicroRNA miR-27a and miR-451 in the regulation of MDR1/P-glycoprotein expression in human cancer cells. Biochem Pharmacol (2008) 76(5):582–8. doi: 10.1016/j.bcp.2008.06.007 PMC2628586 18619946

[118] Sun KX Jiao JW Chen S Liu BL Zhao Y . MicroRNA-186 induces sensitivity of ovarian cancer cells to paclitaxel and cisplatin by targeting ABCB1. J Ovarian Res (2015) 8:80. doi: 10.1186/s13048-015-0207-6 26626440 PMC4667519

[119] Zhao H Yu X Ding Y Zhao J Wang G Wu X . MiR-770-5p inhibits cisplatin chemoresistance in human ovarian cancer by targeting ERCC2. Oncotarget (2016) 7(33):53254–68. doi: 10.18632/oncotarget.10736 PMC5288183 27449101

[120] Ye G Fu G Cui S Zhao S Bernaudo S Bai Y . MicroRNA 376c enhances ovarian cancer cell survival by targeting activin receptor-like kinase 7: implications for chemoresistance. J Cell Sci (2011) 124(Pt 3):359–68. doi: 10.1242/jcs.072223 21224400

[121] Li N Yang L Wang H Yi T Jia X Chen C . MiR-130a and MiR-374a function as novel regulators of cisplatin resistance in human ovarian cancer A2780 cells. PloS One (2015) 10(6):e0128886. doi: 10.1371/journal.pone.0128886 26043084 PMC4456206

[122] Wu H Xiao Z Zhang H Wang K Liu W Hao Q . MiR-489 modulates cisplatin resistance in human ovarian cancer cells by targeting Akt3. Anti-cancer Drugs (2014) 25(7):799–809. doi: 10.1097/CAD.0000000000000107 24686007

[123] Shuang T Wang M Zhou Y Shi C Wang D . NF-κB1, c-rel, and ELK1 inhibit miR-134 expression leading to TAB1 upregulation in paclitaxel-resistant human ovarian cancer. Oncotarget (2017) 8(15):24853–68. doi: 10.18632/oncotarget.15267 PMC5421894 28206956

[124] Chen X Zhou J Li X Wang X Lin Y Wang X . Exosomes derived from hypoxic epithelial ovarian cancer cells deliver microRNAs to macrophages and elicit a tumor-promoted phenotype. Cancer Lett (2018) 435:80–91. doi: 10.1016/j.canlet.2018.08.001 30098399

[125] Bach DH Hong JY Park HJ Lee SK . The role of exosomes and miRNAs in drug-resistance of cancer cells. Int J Cancer (2017) 141(2):220–30. doi: 10.1002/ijc.30669 28240776

[126] Pascut D Pratama MY Vo NVT Masadah R Tiribelli C . The crosstalk between tumor cells and the microenvironment in hepatocellular carcinoma: The role of exosomal microRNAs and their clinical implications. Cancers (2020) 12(4):823. doi: 10.3390/cancers12040823 32235370 PMC7226466

[127] Syn N Wang L Sethi G Thiery JP Goh BC . Exosome-mediated metastasis: From epithelial-mesenchymal transition to escape from immunosurveillance. Trends Pharmacol Sci (2016) 37(7):606–17. doi: 10.1016/j.tips.2016.04.006 27157716

[128] Li I Nabet BY . Exosomes in the tumor microenvironment as mediators of cancer therapy resistance. Mol Cancer (2019) 18(1):32. doi: 10.1186/s12943-019-0975-5 30823926 PMC6397467

[129] Paskeh MDA Entezari M Mirzaei S Zabolian A Saleki H Naghdi MJ . Emerging role of exosomes in cancer progression and tumor microenvironment remodeling. J Hematol Oncol (2022) 15(1):83. doi: 10.1186/s13045-022-01305-4 35765040 PMC9238168

[130] Czystowska-Kuzmicz M Sosnowska A Nowis D Ramji K Szajnik M Chlebowska-Tuz J . Small extracellular vesicles containing arginase-1 suppress T-cell responses and promote tumor growth in ovarian carcinoma. Nat Commun (2019) 10(1):3000. doi: 10.1038/s41467-019-10979-3 31278254 PMC6611910

